# The effect of parathyroid hormone on osteogenesis is mediated partly by osteolectin

**DOI:** 10.1073/pnas.2026176118

**Published:** 2021-06-21

**Authors:** Jingzhu Zhang, Adi Cohen, Bo Shen, Liming Du, Alpaslan Tasdogan, Zhiyu Zhao, Elizabeth J. Shane, Sean J. Morrison

**Affiliations:** ^a^Children’s Research Institute, University of Texas Southwestern Medical Center, Dallas, TX 75235;; ^b^Department of Medicine, Vagelos College of Physicians & Surgeons, Columbia University, New York, NY 10032;; ^c^HHMI, University of Texas Southwestern Medical Center, Dallas, TX 75235;; ^d^Department of Pediatrics, University of Texas Southwestern Medical Center, Dallas, TX 75235

**Keywords:** osteogenesis, parathyroid hormone, osteolectin, stem cells, sclerostin

## Abstract

We previously described a new osteogenic growth factor, osteolectin/Clec11a, which is required for the maintenance of skeletal bone mass during adulthood. Osteolectin binds to Integrin α11 (Itga11), promoting Wnt pathway activation and osteogenic differentiation by leptin receptor^+^ (LepR^+^) stromal cells in the bone marrow. Parathyroid hormone (PTH) and sclerostin inhibitor (SOSTi) are bone anabolic agents that are administered to patients with osteoporosis. Here we tested whether osteolectin mediates the effects of PTH or SOSTi on bone formation. We discovered that PTH promoted *Osteolectin* expression by bone marrow stromal cells within hours of administration and that PTH treatment increased serum osteolectin levels in mice and humans. *Osteolectin* deficiency in mice attenuated Wnt pathway activation by PTH in bone marrow stromal cells and reduced the osteogenic response to PTH in vitro and in vivo. In contrast, SOSTi did not affect serum osteolectin levels and osteolectin was not required for SOSTi-induced bone formation. Combined administration of osteolectin and PTH, but not osteolectin and SOSTi, additively increased bone volume. PTH thus promotes osteolectin expression and osteolectin mediates part of the effect of PTH on bone formation.

## Significance

Only recently have multiple, anabolic, bone-forming agents become available, raising the possibility that osteoporosis might be more effectively treated by combinatorial or sequential treatments rather than single agents. The discovery of osteolectin raised two important questions in this regard. First, what is the epistatic relationship between these agents? Second, would osteolectin have additive effects if combined with another agent? Our discovery that osteolectin mediates part of the effect of parathyroid hormone (PTH) on bone formation identifies a new mechanism by which PTH acts. The observation that PTH and osteolectin have additive effects provides proof-of-principle that combinatorial use can increase osteogenesis.

The maintenance and repair of the skeleton require the generation of new bone cells throughout adult life. Osteoblasts are relatively short-lived cells that are constantly regenerated, partly by skeletal stem cells within the bone marrow (1). The main source of new osteoblasts in adult bone marrow is leptin receptor-expressing (LepR^+^) stromal cells (2–4). These cells include the multipotent skeletal stem cells that give rise to the fibroblast colony-forming cells (CFU-Fs) in the bone marrow (2), as well as restricted osteogenic progenitors (5) and adipocyte progenitors (6–8). LepR^+^ cells are a major source of osteoblasts for fracture repair (2) and growth factors for hematopoietic stem cell maintenance (9–11).

One growth factor synthesized by LepR^+^ cells, as well as osteoblasts and osteocytes, is osteolectin/Clec11a, a secreted glycoprotein of the C-type lectin domain superfamily (5, 12, 13). Osteolectin is an osteogenic factor that promotes the maintenance of the adult skeleton by promoting the differentiation of LepR^+^ cells into osteoblasts. Osteolectin acts by binding to integrin α11β1, which is selectively expressed by LepR^+^ cells and osteoblasts, activating the Wnt pathway (12). Deficiency for either *Osteolectin* or *Itga11* (the gene that encodes integrin α11) reduces osteogenesis during adulthood and causes early-onset osteoporosis in mice (12, 13). Recombinant osteolectin promotes osteogenic differentiation by bone marrow stromal cells in culture and daily injection of mice with osteolectin systemically promotes bone formation.

Osteoporosis is a progressive condition characterized by reduced bone mass and increased fracture risk (14). Several factors contribute to osteoporosis development, including aging, estrogen insufficiency, mechanical unloading, and prolonged glucocorticoid use (14). Existing therapies include antiresorptive agents that slow bone loss, such as bisphosphonates (15, 16) and estrogens (17), and anabolic agents that increase bone formation, such as parathyroid hormone (PTH) (18), PTH-related protein (19), and sclerostin inhibitor (SOSTi) (20). While these therapies increase bone mass and reduce fracture risk, they are not a cure.

PTH promotes both anabolic and catabolic bone remodeling (21–24). PTH is synthesized by the parathyroid gland and regulates serum calcium levels, partly by regulating bone formation and bone resorption (23–25). PTH1R is a PTH receptor (26, 27) that is strongly expressed by LepR^+^ bone marrow stromal cells (8, 28–30). Recombinant human PTH (Teriparatide; amino acids 1 to 34) and synthetic PTH-related protein (Abaloparatide) are approved by the US Food and Drug Administration (FDA) for the treatment of osteoporosis (19, 31). Daily (intermittent) administration of PTH increases bone mass by promoting the differentiation of osteoblast progenitors, inhibiting osteoblast and osteocyte apoptosis, and reducing sclerostin levels (32–35). PTH promotes osteoblast differentiation by activating Wnt and BMP signaling in bone marrow stromal cells (28, 36, 37), although the mechanisms by which it regulates Wnt pathway activation are complex and uncertain (38).

Sclerostin is a secreted glycoprotein that inhibits Wnt pathway activation by binding to LRP5/6, a widely expressed Wnt receptor (7, 8), reducing bone formation (39, 40). Sclerostin is secreted by osteocytes (8, 41), negatively regulating bone formation by inhibiting the differentiation of osteoblasts (41, 42). SOSTi (Romosozumab) is a humanized monoclonal antibody that binds sclerostin, preventing binding to LRP5/6 and increasing Wnt pathway activation and bone formation (43). It is FDA-approved for the treatment of osteoporosis (20, 44) and has activity in rodents in addition to humans (45, 46).

The discovery that osteolectin is a bone-forming growth factor raises the question of whether it mediates the effects of PTH or SOSTi on osteogenesis.

## Results

### PTH Treatment Increases Serum Osteolectin Levels in Patients.

**Fig. 1.**
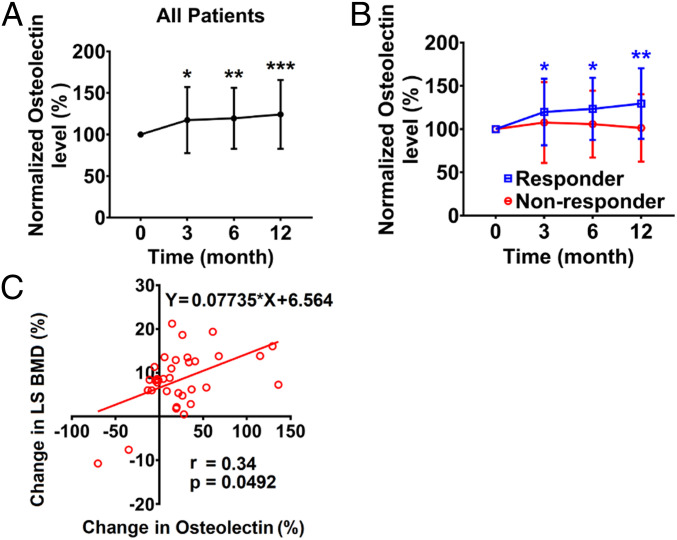
Serum osteolectin levels in premenopausal women with idiopathic osteoporosis after receiving PTH treatment. Osteolectin levels were measured in blinded patient samples at baseline and then at 3, 6, and 12 mo after initiating Teriparatide treatment. The levels are shown as a percentage of the level in the same patient at baseline. (*A*) Serum osteolectin levels in all patients (*n* = 40). (*B*) Serum osteolectin levels in responders (*n* = 32) and nonresponders (*n* = 8) to Teriparatide. These groups were prespecified based on a prior study (47). (*C*) Correlation between the percentage change in lumbar spine bone mineral density and the percentage change in serum osteolectin levels in all patients after 12 mo of Teriparatide treatment (*n* = 34). All data represent mean ± SD. Statistical significance was assessed using two-sided, one-sample Wilcoxon signed rank tests for time points compared to baseline followed by multiple comparisons adjustment using the Holm–Sidak method (*A*), two-way ANOVAs followed by the Holm–Sidak method (*B*), or a simple linear regression with the Spearman correlation test (*C*); *P < 0.05, **P < 0.01, ***P < 0.001.

To test if Osteolectin levels are altered by PTH treatment, we quantified osteolectin levels in serum samples from patients treated daily with Teriparatide, a polypeptide containing the first 34 amino acids of PTH (18). These samples were collected from patients in a phase II clinical trial of Teriparatide for the treatment of idiopathic osteoporosis in premenopausal women (NCT01440803) (47). The patients were divided into two prespecified groups according to whether or not they exhibited a significant increase in lumbar spine bone mineral density after 12 mo of PTH treatment. In responders (*n* = 32), lumbar spine bone mineral density increased above the manufacturer-provided least-significant change (0.026 g/cm^2^), whereas nonresponders (*n* = 8) exhibited an absolute change in lumbar spine bone mineral density below the manufacturer-provided least-significant change (16). We measured osteolectin levels in the serum of these prespecified groups while blinded to sample identity. We observed a significant increase in serum osteolectin levels at 3, 6, and 12 mo after initiating PTH treatment in all patients relative to pretreatment levels in the same patients (Fig. 1*A*). The responders also exhibited a significant increase in serum osteolectin levels at 3, 6, and 12 mo, but not the nonresponders (Fig. 1*B*). The differences between responders and nonresponders in serum osteolectin levels were not statistically significant; however, there was a statistically significant correlation between the percentage change in lumbar spine bone mineral density and the percentage change in serum osteolectin levels in all patients after 12 mo of PTH treatment (Fig. 1*C*). Increased bone formation after PTH treatment in humans is thus associated with an increase in serum osteolectin levels.

### Osteolectin Mediates Part of the Effect of PTH on Bone Volume.

**Fig. 2.**
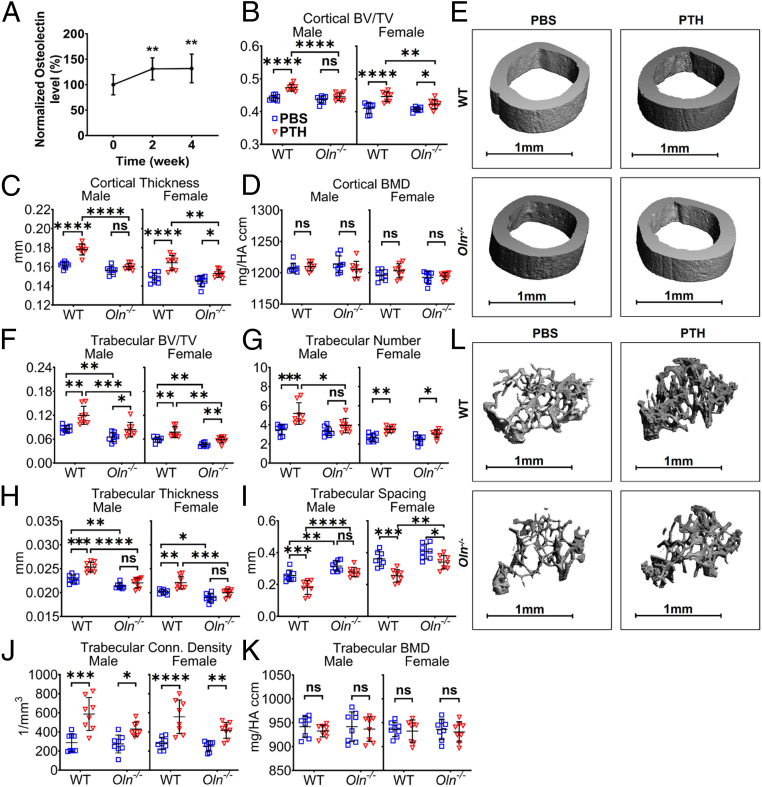
Osteolectin mediates part of the effect of PTH on bone volume. (*A*) Serum osteolectin levels were measured in 2-mo-old wild-type (WT) mice treated with daily injections of PTH for 2 or 4 wk. Values in each mouse are presented as a percentage of baseline levels in the same mouse prior to treatment (*n* = 5 male and 5 female mice per group). (*B*–*D*) MicroCT analysis of cortical bone volume/total volume (BV/TV) (*B*), cortical thickness (*C*), and cortical bone mineral density (BMD) (*D*) in the midfemur diaphysis of *Osteolectin*-deficient (*Oln*^*−/−*^) or control (WT) mice treated with PBS or PTH for 4 wk. (*E*) Representative microCT images of cortical bone in the midfemur diaphysis of male *Oln*^*−/−*^ and control mice treated with PBS or PTH for 4 wk. (*F*–*K*) MicroCT analysis of the trabecular bone volume/total volume (*F*), trabecular number (*G*), trabecular thickness (*H*), trabecular spacing (*I*), trabecular connectivity density (*J*), and trabecular bone mineral density (*K*) in the distal femur metaphysis of *Oln*^*−/−*^ and control mice treated with PBS or PTH for 4 wk. (*L*) Representative microCT images of trabecular bone in the distal femur metaphysis of male *Oln*^*−/−*^ and control mice treated with PBS or PTH for 4 wk. *B*–*K* represent data from *n* = 8 male or female mice per treatment. All data represent mean ± SD. Statistical significance was assessed using one-way ANOVAs followed by Dunnett’s multiple comparisons tests (*A*) or three-way ANOVAs followed by Sidak’s multiple comparisons tests (*P < 0.05; **P < 0.01; ***P < 0.001; ****P < 0.0001; ns, not significant).

To test if PTH treatment increased serum osteolectin levels in mice, 2-mo-old wild-type mice were administered daily subcutaneous injections of Teriparatide for 2 or 4 wk. Consistent with the results in patients, PTH treatment also significantly increased serum osteolectin levels in mice (Fig. 2*A*). We did not detect an increase in serum osteolectin levels after PTH treatment for less than 24 h (*SI Appendix*, Fig. S1*A*), suggesting that longer-term treatment is required to observe a change in serum osteolectin levels.

To test if osteolectin mediates the effect of PTH on bone formation, we treated *Osteolectin*-deficient (*Oln*^*−/−*^) and sex-matched littermate control mice with daily injections of PTH for 4 wk. PTH treatment significantly increased cortical bone volume and cortical thickness in control mice but had little or no effect in *Osteolectin*-deficient mice (Fig. 2 *B*–*E*). PTH treatment also significantly increased trabecular bone volume, trabecular number, and trabecular thickness in control mice but had significantly reduced effects in *Osteolectin*-deficient mice (Fig. 2 *F*–*H*). Consistent with this, we observed a significant reduction in trabecular spacing in control mice, but a significantly smaller effect in *Osteolectin*-deficient mice (Fig. 2*I*). PTH treatment increased trabecular connectivity density in male and female *Osteolectin*-deficient mice, although there was a trend toward larger effects in control mice (Fig. 2*J*). *Osteolectin* deficiency thus reduced, but did not completely eliminate, the effects of PTH treatment on cortical and trabecular bone volume.

PTH treatment significantly increased serum procollagen type 1 N-terminal propeptide (P1NP) levels, a marker of bone formation, in both *Osteolectin*-deficient and control mice, but the effect of PTH was significantly larger in the control mice (*SI Appendix*, Fig. S1*B*). PTH treatment also significantly increased serum C-telopeptide of type I collagen (CTX1) levels, a marker of bone resorption, in both *Osteolectin*-deficient and control mice (*SI Appendix*, Fig. S1*C*). There was no effect of *Osteolectin* deficiency on CTX1 levels. Serum SOST levels were not significantly affected by PTH treatment or *Osteolectin* deficiency (*SI Appendix*, Fig. S1*D*). Consistent with a prior study (13), these data suggest that *Osteolectin* deficiency reduced the effect of PTH on bone formation without affecting bone resorption.

We previously reported that osteolectin administration to *Osteolectin*-deficient or control mice had no effect on CFU-F frequency in the bone marrow (13). Osteolectin administration and *Osteolectin* deficiency also had no effect on the frequency of osteolectin^+^LepR^+^ periarteriolar osteogenic progenitors (5) in the bone marrow (*SI Appendix*, Fig. S1 *E* and *F*). Therefore, we have no evidence that osteolectin promotes the expansion of an osteogenic progenitor, though it may accelerate the differentiation of skeletal stem/progenitor cells.

### Osteolectin Is Not Required for the Effects of SOSTi on Bone Volume.

**Fig. 3.**
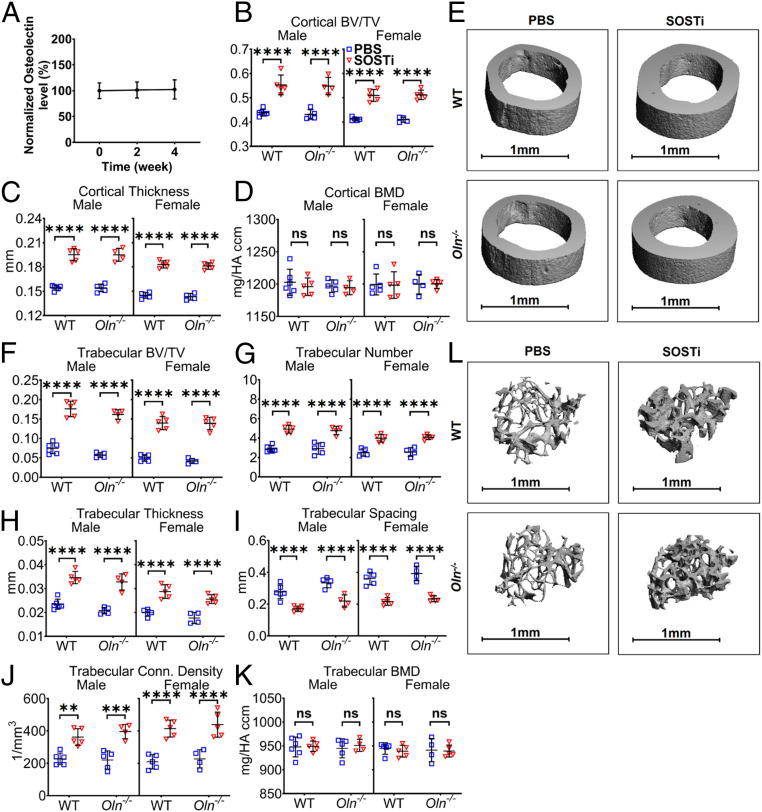
Osteolectin is not required for the effect of SOSTi on bone volume. (*A*) Serum osteolectin levels were measured in 2-mo-old wild-type (WT) mice treated with biweekly injections of SOSTi for 2 or 4 wk. Values in each mouse are presented as a percentage of baseline levels in the same mouse prior to treatment (*n* = 5 male and 5 female mice per group). (*B*–*D*) MicroCT analysis of the cortical bone volume/total volume (*B*), cortical thickness (*C*), and cortical bone mineral density (*D*) in the midfemur diaphysis of *Osteolectin*-deficient (*Oln*^*−/−*^) and control (WT) mice treated with PBS or SOSTi for 4 wk. (*E*) Representative microCT images of cortical bone in the midfemur diaphysis of male *Oln*^*−/−*^ and control mice treated with PBS or SOSTi for 4 wk. (*F*–*K*) MicroCT analysis of the trabecular bone volume/total volume (*F*), trabecular number (*G*), trabecular thickness (*H*), trabecular spacing (*I*), trabecular connectivity density (*J*), and trabecular bone mineral density (*K*) in the distal femur metaphysis of *Oln*^*−/−*^ and control mice treated with PBS or SOSTi for 4 wk. (*L*) Representative microCT images of trabecular bone in the distal femur metaphysis of male *Oln*^*−/−*^ and control mice treated with PBS or SOSTi for 4 wk. *B*–*K* represent data from *n* = 4 to 6 male mice and 4 to 5 female mice per treatment. All data represent mean ± SD. Statistical significance was assessed using one-way ANOVAs followed by Dunnett’s multiple comparisons tests (*A*), three-way ANOVAs followed by Sidak’s multiple comparisons tests (*B*–*D*, *F*–*I*, and *K*), or one-way ANOVAs followed by Sidak’s multiple comparisons tests (*J*) (**P < 0.01; ***P < 0.001; ****P < 0.0001).

To test if treatment with SOSTi influences serum osteolectin levels, we treated 2-mo-old wild-type mice with biweekly subcutaneous injections of SOSTi for 2 or 4 wk. In contrast to PTH, we observed no effect of SOSTi treatment on serum osteolectin levels (Fig. 3*A*). To test if osteolectin mediates the effect of SOSTi on bone volume, *Osteolectin*-deficient and sex-matched littermate control mice were administered biweekly injections of SOSTi for 4 wk. Both wild-type and *Osteolectin*-deficient mice exhibited similarly robust increases in cortical bone volume and cortical thickness after SOSTi treatment (Fig. 3 *B*–*E*). We also observed robust increases in trabecular bone volume, trabecular number, trabecular thickness, and trabecular connectivity density in both male and female wild-type and *Osteolectin*-deficient mice (Fig. 3 *F*–*L*). Consistent with this, we observed a significant reduction in trabecular spacing in both male and female wild-type and *Osteolectin*-deficient mice (Fig. 3*I*). These data suggest that osteolectin is not required for SOSTi to increase cortical or trabecular bone volume.

### Osteolectin Contributes to Wnt Pathway Activation by PTH.

**Fig. 4.**
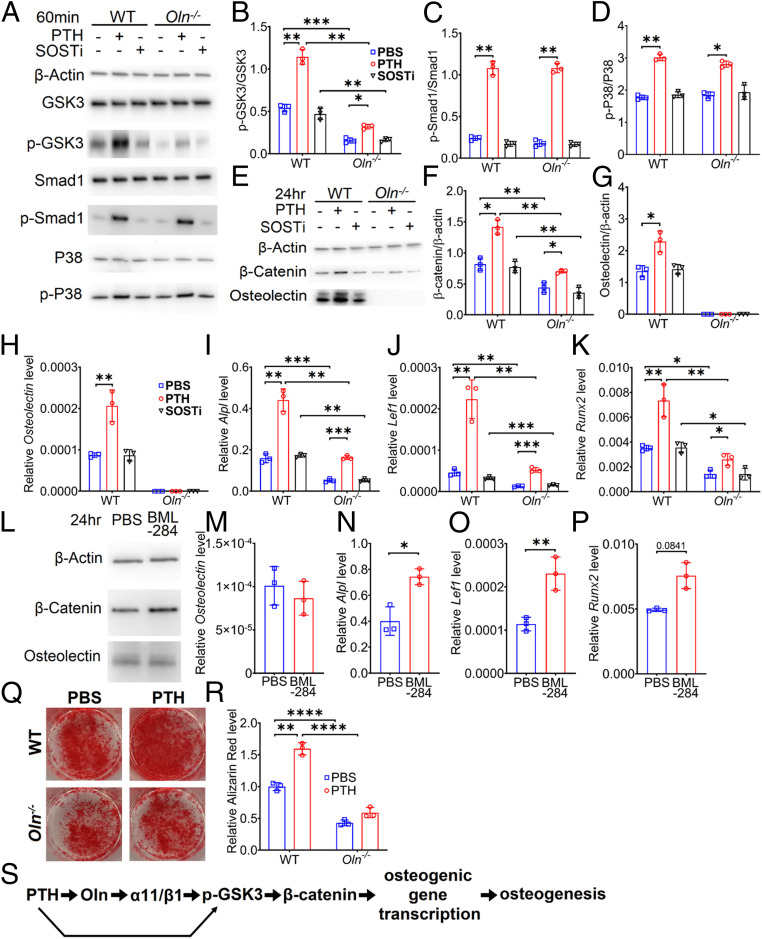
Osteolectin deficiency attenuates PTH-induced Wnt signaling. Bone marrow stromal cells cultured from *Osteolectin*-deficient (*Oln*^*−/−*^) or littermate control (WT) mice were treated with PTH (10 nM), SOSTi (10 ng/mL), or PBS (control) in osteogenic differentiation medium. (*A*–*D*) Bone marrow stromal cells were lysed 60 min after treatment and immunoblotted for β-actin, GSK3, phospho-GSK3 (p-GSK3), Smad1, phospho-Smad1 (p-Smad1), P38, and phospho-P38 (p-P38) (representative of three independent experiments). (*B*) Phospho-GSK3 levels normalized to total GSK3 levels. (*C*) Phospho-Smad1 levels normalized to total Smad1 levels. (*D*) Phospho-P38 levels normalized to total P38 levels. (*E*–*G*) Bone marrow stromal cells were lysed 24 h after treatment and immunoblotted for β-actin, β-catenin, and osteolectin (representative of three independent experiments). (*F*) β-Catenin levels normalized to β-actin levels. (*G*) Osteolectin levels normalized to β-actin levels. (*H–K*) Bone marrow stromal cells were lysed 3 d after treatment and analyzed by qRT-PCR to assess *Osteolectin* (*H*) and Wnt target gene transcript levels including *Alpl* (*I*), *Lef1* (*J*), and *Runx2* (*K*). (*L*–*P*) *C*ontrol bone marrow stromal cells cultured in osteogenic differentiation medium were treated with PBS or BML-284, a Wnt agonist, then lysed 24 h later and immunoblotted for β-actin, β-catenin, and osteolectin (*L*) or analyzed 3 d later by qRT-PCR to assess *Osteolectin* (*M*) or Wnt target gene transcript levels including *Alpl* (*N*), *Lef1* (*O*), and *Runx2* (*P*) (*n* = 3 independent experiments). (*Q* and *R*) *Oln*^*−/−*^ or control bone marrow stromal cells cultured in osteogenic differentiation medium were treated with PTH or PBS for 14 d and stained with Alizarin red S (*n* = 3 independent experiments). (*S*) The effect of PTH on Wnt pathway activation and osteogenic differentiation are mediated partly by osteolectin (Oln). All data represent mean ± SD. Statistical significance was assessed using paired sample two-way ANOVAs followed by Dunnett’s (comparing treatments in *B*–*D*, *F*–*K*, and *R*), Holm–Sidak’s (comparing genotypes in *B*–*D* and *F*–*K*) or Sidak’s (*M*–*P* and comparing genotypes in *R*) multiple comparisons tests (*P < 0.05; **P < 0.01; ***P < 0.001; ****P < 0.0001).

To test whether *Osteolectin* deficiency affects the ability of PTH to promote Wnt pathway activation, we adherently cultured bone marrow stromal cells from *Osteolectin*-deficient and sex-matched littermate control mice. We treated the cells with PTH, SOSTi, or vehicle control. Total GSK3, Smad1, and P38 levels were similar in *Osteolectin*-deficient and control stromal cells, and were not affected by PTH or SOSTi treatment (Fig. 4*A*). Consistent with the observation that osteolectin is secreted by bone marrow stromal cells in culture and promotes autocrine Wnt pathway activation (12), phosphorylated GSK3 levels were higher in control cells than in *Osteolectin*-deficient cells (Fig. 4 *A* and *B*). PTH treatment increased the levels of phosphorylated GSK3 (Ser21/9) to a greater extent in control cells than in *Osteolectin*-deficient cells (Fig. 4 *A* and *B*), suggesting Wnt pathway activation by PTH was attenuated in the absence of osteolectin. In contrast, PTH treatment increased phosphorylated Smad1 and P38 levels to similar extents in both *Osteolectin*-deficient and control cells (Fig. 4 *A*, *C*, and *D*), suggesting a similar ability to promote BMP pathway activation (28, 48).

SOSTi did not increase the levels of phosphorylated GSK3, Smad1, or P38 in either *Osteolectin*-deficient or control cells (Fig. 4 *A*–*D*). This may reflect the absence of sclerostin in bone marrow stromal cell cultures before they undergo osteogenic differentiation (8, 41).

GSK3 phosphorylation inhibits the degradation of β-catenin, promoting the transcription of Wnt pathway target genes (15, 17, 49). Consistent with autocrine Wnt pathway activation by osteolectin in control cell cultures (12) (Fig. 4 *A* and *B*), β-catenin levels were higher in control cells than in *Osteolectin*-deficient cells (Fig. 4 *E* and *F*). Moreover, after 24 h, PTH treatment increased the levels of β-catenin to a significantly greater extent in control as compared to *Osteolectin*-deficient cells (Fig. 4 *E* and *F*), further suggesting that Wnt pathway activation by PTH was attenuated in the absence of osteolectin.

Consistent with the effects of PTH on serum osteolectin levels (Figs. 1*A* and 2*A*), PTH treatment increased osteolectin production by bone marrow stromal cells (Fig. 4 *E*, *G*, and *H*). This may not have been a consequence of Wnt pathway activation as treatment of control stromal cells with a small molecule that stabilizes β-catenin (Fig. 4*L*) promoted the transcription of other Wnt pathway targets but not *Osteolectin* (Fig. 4 *M*–*P*). We also treated bone marrow stromal cells with PTH for 0.5, 1, 2, 4, 8, or 24 h. We observed a significant increase in *Osteolectin* transcript levels at 8 and 24 h after PTH treatment (*SI Appendix*, Fig. S2*C*). We observed no significant change in the numbers of live cells in these cultures and cell death was rare at all time points (*SI Appendix*, Fig. S2 *A*–*D*). PTH thus acutely promoted *Osteolectin* expression by bone marrow stromal cells.

PTH promoted osteogenic differentiation by control cells, but not by *Osteolectin*-deficient cells, in culture (Fig. 4 *Q* and *R*). Consistent with this, and the increased Wnt pathway activation in control as compared to *Osteolectin*-deficient cells (Fig. 4 *A*, *B*, *E*, and *F*), control cells exhibited significantly higher transcription of the Wnt pathway target genes *Alpl* (50), *Lef1* (51), and *Runx2* (52) as compared to *Osteolectin*-deficient cells (Fig. 4 *I*–*K*). Moreover, PTH treatment increased the transcription of Wnt pathway target genes to a greater extent in control than *Osteolectin*-deficient cells (Fig. 4 *I*–*K*), further indicating that Wnt pathway activation by PTH was attenuated in the absence of osteolectin. Treatment with SOSTi did not significantly affect the transcription of Wnt target genes (Fig. 4 *I*–*K*), consistent with the lack of Wnt pathway activation by SOSTi in these cultures (Fig. 4 *A*, *B*, *E*, and *F*).

To test if osteolectin is acutely required by bone marrow stromal cells to exhibit increased Wnt pathway activation in response to PTH treatment, we cultured bone marrow stromal cells from *UBC*^*CreER*^*;Osteolectin*^*flox/flox*^ mice and treated them with tamoxifen for 2 d in culture to conditionally delete *Osteolectin* (*SI Appendix*, Fig. S2 *E*–*G*). Conditional deletion of *Osteolectin* was confirmed at the protein and mRNA levels (*SI Appendix*, Fig. S2 *H* and *I*). PTH treatment increased β-catenin protein levels and *Alpl*, *Lef1*, and *Runx2* transcript levels to a significantly greater extent in control as compared to *Osteolectin*-deficient cells (*SI Appendix*, Fig. S2 *H*–*L*). Thus, acute deletion of *Osteolectin* attenuated Wnt pathway activation and osteogenic differentiation (*SI Appendix*, Fig. S2 *M* and *N*) in response to PTH treatment (Fig. 4S).

### PTH and Osteolectin Additively Increase Bone Volume.

**Fig. 5.**
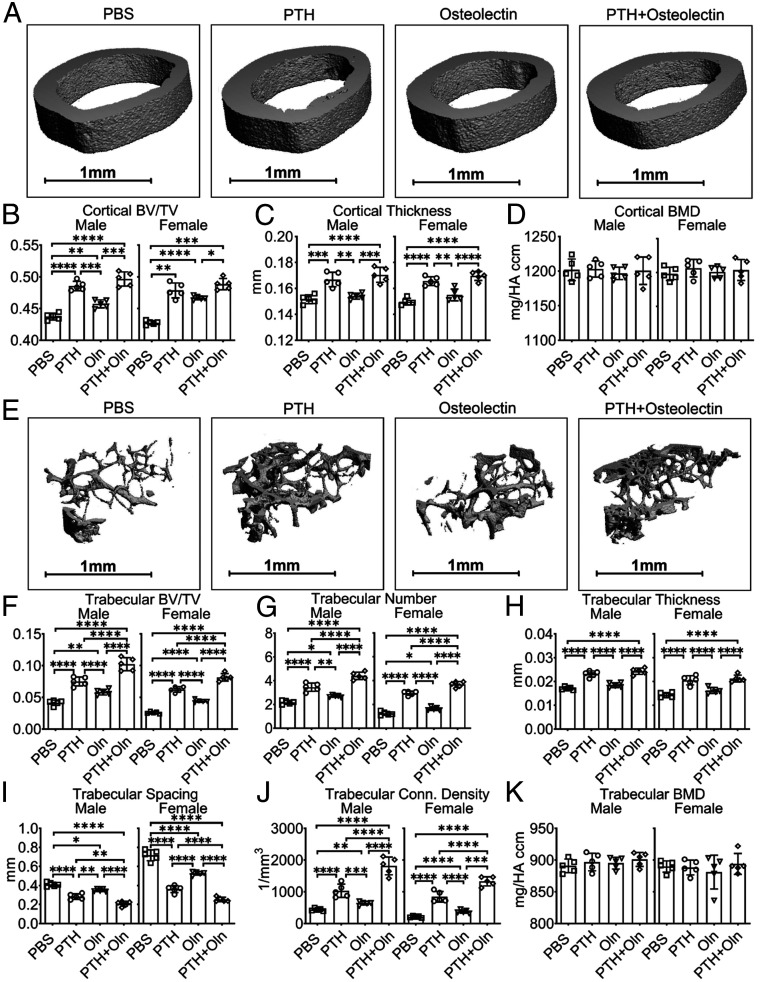
PTH and osteolectin additively increase trabecular bone volume. (*A*) Representative microCT images of cortical bone in the midfemur diaphysis of male wild-type (WT) mice treated with PBS, PTH, osteolectin (Oln), or the combination of PTH plus osteolectin for 3 mo. (*B*–*D*) MicroCT analysis of the cortical bone volume/total volume (*B*), cortical thickness (*C*), and cortical bone mineral density (*D*) in the midfemur diaphysis of these mice. (*E*) Representative microCT images of trabecular bone in the distal femur metaphysis of male mice. (*F*–*K*) MicroCT analysis of the trabecular bone volume/total volume (*F*), trabecular number (*G*), trabecular thickness (*H*), trabecular spacing (*I*), trabecular connectivity density (*J*), and trabecular bone mineral density (*K*) in the distal femur metaphysis of these mice. For all experiments, *n* = 5 mice per group. All data represent mean ± SD. Statistical significance was assessed using one-way ANOVAs (*C*, *F*, *G*, and *J*) or two-way ANOVAs (*D*, *H*, *I*, and *K*) followed by Tukey’s multiple comparisons tests (*P < 0.05; **P < 0.01; ***P < 0.001; ****P < 0.0001).

To test if PTH and osteolectin had additive effects on bone parameters, 2-mo-old wild-type mice were administered daily injections of PTH, osteolectin, PTH+osteolectin, or PBS (control) for 3 mo. All three treatments significantly increased cortical bone volume relative to control mice, though PTH treatment had larger effects on cortical bone volume and thickness than this dose of osteolectin (Fig. 5 *A*–*D*). For cortical bone parameters, the combination of PTH and osteolectin did not significantly differ from PTH alone. All three treatments also significantly increased trabecular bone volume, trabecular number, and trabecular connectivity density relative to control mice and reduced trabecular spacing (Fig. 5 *E*–*K*). While each of osteolectin and PTH alone had significant effects on most trabecular bone parameters, the effects of PTH were significantly greater than this dose of osteolectin. Nonetheless, the combination of osteolectin and PTH had significantly greater effects on most trabecular bone parameters (the only exception was trabecular thickness) than PTH alone or osteolectin alone (Fig. 5 *E*–*K*). Recombinant PTH and osteolectin thus have additive effects on trabecular bone parameters.

We also tested if SOSTi and osteolectin had additive effects on bone parameters by administering SOSTi, osteolectin, SOSTi+osteolectin, or PBS to 2-mo-old mice for 1 mo. Treatment with SOSTi significantly increased cortical bone volume and thickness, whereas osteolectin treatment for only 1 mo did not (*SI Appendix*, Fig. S3 *A*–*D*). The combination of SOSTi and osteolectin did not significantly differ from SOSTi alone. All three treatments significantly increased trabecular bone volume and trabecular number while reducing trabecular spacing relative to control mice (*SI Appendix*, Fig. S3 *E*–*K*). The effects of SOSTi were significantly greater than the effects of osteolectin. In contrast to the additive effects of PTH and osteolectin, the combination of osteolectin and SOSTi did not significantly differ from SOSTi alone.

## Discussion

We found that osteolectin mediates part of the effect of PTH, but not SOSTi, on Wnt pathway activation and bone formation. One possibility is that osteolectin and PTH both increase the number of osteogenic progenitors or accelerate their differentiation into osteoblasts while SOSTi acts on more differentiated cells. The PTH receptor, PTH1R, is more highly expressed by LepR^+^ bone marrow stromal cells than osteoblasts (8), raising the possibility that it acts mainly by promoting the differentiation of undifferentiated cells. Osteolectin also promotes the differentiation of LepR^+^ cells and other undifferentiated stromal cells (12, 13). In contrast, the sclerostin receptor, LRP5/6, is more highly expressed by osteoblasts than LepR^+^ cells (7, 8), raising the possibility that sclerostin acts primarily on more differentiated cells. Recombinant osteolectin and PTH may thus both promote Wnt signaling in undifferentiated cells, such that when combined, they activate the pathway more than either factor alone. In contrast, SOSTi may act primarily on mature osteoblasts and quiescent bone-lining cells (45).

Although osteolectin had less potent effects on bone parameters than PTH or SOSTi, this may not reflect a fundamental limitation of osteolectin. No structure/function or dosage optimization studies have been performed with osteolectin. Higher doses of osteolectin might have more dramatic effects. Variant forms of the protein or altered dosing schedules could also have more potent effects.

Patients commonly become less responsive to anabolic, bone-forming agents after 1 y of treatment and may stop responding entirely after 3 y (53–55). This may be a consequence of depletion of osteogenic stem/progenitor cells. An important issue for future studies will be to test whether sequential treatment with different anabolic agents can sustain osteogenic responses for a longer period of time. That is, do patients retain the ability to respond to a second anabolic agent after they stop responding to a first agent? If so, the sequential use of multiple anabolic agents could sustain osteogenesis for longer.

While PTH (Teriparatide) and SOSTi (Romosozumab) are FDA-approved for use in patients with osteoporosis (20, 31), both drugs have risks. PTH regulates calcium metabolism (23–25), mild hypercalcaemia was observed in 11% of patients in a clinical trial (18), and PTH should not be used in patients with hypercalcaemia (56). A study in rats found that long-term PTH administration induced osteosarcoma (57). Although no evidence of osteosarcoma has been observed in humans treated with PTH (18, 58), PTH should be avoided in patients with elevated risk of osteosarcoma and a history of cancer, including adolescents in whom the epiphyses have not yet closed, those with Paget’s disease or prior skeletal radiation, and patients with unexplained increases in alkaline phosphatase (59). SOSTi was found to modestly increase the risk of cardiovascular events (60), raising concerns about the use of SOSTi in patients with cardiovascular conditions.

The safety of long-term osteolectin administration has not been tested but *Osteolectin*-deficient mice have no known phenotypes other than reduced bone formation during adulthood (13). Moreover, the osteolectin receptor, α11 integrin, is widely expressed by osteolineage cells but rarely by nonosteolineage cells. These observations raise the possibility that osteolectin treatment might have limited effects on nonosteolineage cells and a favorable safety profile.

We observed increased serum osteolectin levels after PTH treatment in premenopausal women with idiopathic osteoporosis, but whether similar effects would be observed in older postmenopausal women remains unclear. We also demonstrated the osteogenic effect of recombinant osteolectin on cortical and trabecular bone in young adult mice (Fig. 5). However, whether osteolectin has similar effects in older mice requires future study. Further exploration of the functions of osteolectin in skeletal homeostasis at different ages could reveal new mechanisms that influence the development of osteoporosis during aging.

## Materials and Methods

### Mice.

*Osteolectin*^*−/−*^ (*Clec11a*^*−/−*^) (13) mice were backcrossed at least six times onto a C57BL/*K*_a_ background and compared with sex-matched wild-type littermate controls. *Osteolectin*^*tdtomato/+*^ and *Osteolectin*^*tdtomato/−*^ mice (5) were also backcrossed at least six times onto a C57BL/*K*_a_ background and compared with sex-matched littermate controls. To generate *Osteolectin*^*flox*^ mice, CleanCap Cas9 mRNA (TriLink) and single-guide RNAs (transcribed using MEGAshortscript Kit [Ambion], purified using the MEGAclear Kit [Ambion]), and recombineering single-strand DNA were microinjected into C57BL/*K*_a_ zygotes. Chimeric mice were genotyped by restriction fragment-length poly-morphism analysis and confirmed by sequencing of the targeted allele. Founders were mated with C57BL/*K*_a_ mice to obtain germline transmission then backcrossed with wild-type C57BL/*K*_a_ mice for at least three generations prior to analysis. All procedures were approved by the University of Texas Southwestern Institutional Animal Care and Use Committee (protocol number 2017-101896).

### PTH, Osteolectin, and SOSTi Treatments.

In some experiments, mice were treated with daily injections of 40 µg/kg human PTH (amino acids 1 to 34, APExBIO), daily injections of 50 µg/kg recombinant mouse osteolectin (13), biweekly injections of 25 mg/kg SOSTi (Romosozumab, Amgen), or 100 µL phosphate-buffered saline (PBS) as vehicle control. Romosozumab is a humanized monoclonal antibody against sclerostin that has been approved by the FDA to treat osteoporosis in postmenopausal women. This antibody also has activity against sclerostin in rodents (45, 46). All injections were subcutaneous.

### Genotyping Primers.

To genotype *Osteolectin*^*−/−*^ and control mice, the following primers were used: 5′-TTT GGG TGC TGG GAA GCC C-3′ and 5′-TTG CAC TGA GTC GCG GGT G-3′, 5′-GAG GAA GAG GAA ATC ACC ACA GC-3′ and 5′-TTG CAC TGA GTC GCG GGT G-3′. To genotype *UBC*^*CreER*^*;Osteolectin*^*flox/flox*^ and control mice, the following primers were used: 5′-ATG TCC AAT TTA CTG ACC GTA CA-3′ and 5′-CGC ATA ACC AGT GAA ACA GCA TT-3′ for *Cre*, and 5′-CAG CTG GAT GCA GGC AGC CTG GCT CT-3′ and 5′- AGG TGC TGT GGT GAT TTC CTC TTC CTC T-3′ for *Osteolectin*^*flox*^.

### Recombinant Osteolectin Purification.

As previously described (12, 13), mouse *Osteolectin* cDNA was cloned into pcDNA3 vector (Invitrogen) containing a C-terminal 1XFlag-tag and transfected into HEK293 cells with Lipofectamine 2000 (Invitrogen) then stable clones were selected using 1 mg/mL G418 (Sigma). Stable clones with high osteolectin expression were cultured in DMEM plus 10% FBS (Sigma), and 1% penicillin/streptomycin (Invitrogen). Culture medium was collected every 2 d, centrifuged to eliminate cellular debris, and stored with 1 mM phenylmethylsulfonyl fluoride at 4 °C to inhibit protease activity. One liter of culture medium was filtered through a 0.2-µm membrane to further eliminate cellular debris (Nalgene) before being loaded onto a chromatography column containing 2 mL anti-FLAG M2 Affinity Gel (Sigma) with a flow rate of 1 mL/min. The column was sequentially washed using 20 mL of high salt buffer (20 mM Tris⋅HCl, 300 mM KCl, 10% Glycerol, 0.2 mM EDTA) followed by 20 mL of low salt buffer (20 mM Tris⋅HCl, 150 mM KCl, 10% Glycerol, 0.2 mM EDTA) and finally 20 mL of PBS. The FLAG-tagged osteolectin was then eluted from the column using 10 mL 3× FLAG peptide (100 μg/mL) in PBS or protein storage buffer (50 mM Hepes, 150 mM NaCl and 10% glycerol, pH = 7.5). Eluted protein was concentrated using Amicon Ultra-15 Centrifugal Filter Units (Ultracel-10K, Millipore), then quantitated by SDS/PAGE and colloidal blue staining (Invitrogen) and stored at −80 °C.

### MicroCT Analysis.

MicroCT analysis was performed using the settings described previously (12, 13). As described previously (61), mouse femurs were dissected, fixed overnight in 4% paraformaldehyde (Thermo Fisher Scientific) and stored in 70% ethanol at 4 °C. Femurs were scanned at an isotropic voxel size of 3.5 μm and 7 μm, respectively, with peak tube voltage of 55 kV and current of 0.145 mA (μCT 35; Scanco). A three-dimensional Gaussian filter (s = 0.8) with a limited, finite filter support of one was used to suppress noise in the images, and a threshold of 263 to 1,000 was used to segment mineralized bone from air and soft tissues. Trabecular bone parameters were measured in the distal metaphysis of the femurs. The region of interest was selected from below the distal growth plate where the epiphyseal cap structure completely disappeared and continued for 100 slices toward the proximal end of the femur. Contours were drawn manually a few voxels away from the endocortical surface to define trabecular bone in the metaphysis. Cortical bone parameters were measured by analyzing 100 slices in middiaphysis femurs.

### ELISAs.

As previously described (13), mouse blood serum was diluted 1:1 using 2× PBS and 100 μL of the diluted serum was coated on each well of a 96-well ELISA plate (COSTAR 96-well EIA/RIA stripwell plate) at 4 °C for 16 h. The plate was then washed three times with washing buffer (PBS with 0.1% Tween-20), blocked with 300 μL ELISA Blocking Buffer (Thermo, N502) for 2 h at room temperature, and washed three times with washing buffer. Antiosteolectin antibody (1 μg/mL diluted in 100 μL of PBS with 0.1% Tween-20 per well) was then added and incubated at room temperature for 2 h, washed three times with washing buffer, followed by incubating with HRP-conjugated donkey anti-goat IgG secondary antibody (0.8 μg/mL diluted in 100 μL of PBS with 0.1% Tween-20 per well) at room temperature for 1 h. After washing three times, 100 μL of SureBlue TMB microwell peroxidase substrate was added to each well and incubated at room temperature in the dark for 15 min. Finally, 100 μL of the TMB stop solution was added into each well and the optical density was measured at 450 nm. Serum P1NP levels were determined using the Procollagen type 1 N-terminal Propeptide (P1NP) ELISA Kit (Antibodies-online, ABIN415750). Serum CTX1 levels were determined using the Mouse CTX1 ELISA Kit (Novus Biologicals, NBP2-69074). SOST levels were determined using the mouse/rat SOST/Sclerostin Quantikine ELISA Kit (R&D Systems, MSST00).

### Measurement of Human Serum Osteolectin Levels.

Serum samples were obtained from 40 female patients with premenopausal idiopathic osteoporosis in a phase IIb trial (NCT01440803) (47). All patients received PTH (Teriparatide, PTH amino acids 1 to 34; Eli Lily), 20 µg subcutaneous injection daily for 12 mo. Fasting, morning serum samples were collected at baseline before initiating Teriparatide treatment and then at 3, 6, and 12 mo after initiating treatment. Based on the presence or absence of lumbar spine bone density responses in these patients, they were categorized as responders or nonresponders (47). We measured serum osteolectin levels in these samples with the Bio-Plex Pro Human Cytokine SCGF-β Set (Bio-Rad), which detects native osteolectin, using the Bio-Plex 200 System (Bio-Rad). To quantitate human osteolectin levels, known concentrations of recombinant human osteolectin/Clec11a/SCGF-α (R&D Systems) were used to create a standard curve with the Bio-Plex system. Osteolectin levels were then measured in samples in a manner blinded to sample identity by J.Z. and then sent to A.C. for unblinding and analysis.

### Bone Marrow Stromal Cell Cultures.

As previously described (12, 13), mouse femurs and tibias were cut at both ends to flush out intact bone marrow plugs. These flushed plugs and crushed bone metaphyses were subjected to two rounds of enzymatic digestion in prewarmed digestion buffer containing 3 mg/mL type I collagenase (Worthington), 4 mg/mL dispase (Roche Diagnostic), and 1 U/mL DNase I (Sigma) in HBSS with calcium and magnesium, at 37 °C for 15 min in each round of digestion. During each round of digestion, the suspension was vortexed six times for 10 s each time at speed level three using a Vortex-Genie 2 to promote more complete dissociation. Dissociated cells were transferred into a tube with staining medium (HBSS without calcium and magnesium +2% fetal bovine serum) and 2 mM EDTA to stop the digestion. Cells were then centrifuged, resuspended in staining medium, and passed through a 90-μm nylon mesh to generate a single-cell suspension.

The freshly dissociated bone marrow cell suspensions were cultured adherently at clonal density in six-well plates (5 × 10^5^ cells per well) or 10-cm plates (5 × 10^6^ cells per dish) with DMEM (Gibco) plus 20% fetal bovine serum (Sigma F2442), 10 mM ROCK inhibitor (Y-27632, Selleck), and 1% penicillin/streptomycin (Invitrogen) at 37 °C in gas-tight chambers (Billups-Rothenberg) flushed with 1% O_2_ and 6% CO_2_ (balance N_2_) to maintain a more physiological oxygen level that promoted survival and proliferation (62). The culture dish was rinsed with HBBS without calcium and magnesium and replenished with fresh medium on the second day after plating to wash out contaminating macrophages. Cultures were then maintained in the gas-tight chambers that were flushed daily for 1 min with the low oxygen gas mix (1% O_2_, 6% CO_2_, balance N_2_). The culture medium was changed every 4 d. To conditionally delete *Osteolectin* from bone marrow stromal cells from *UBC*^*CreER*^*;Osteolectin*^*flox/flox*^ mice, 200 nM 4-hydroxytamoxifen was added to the culture medium for 2 d.

To perform experiments with the bone marrow stromal cells, they were then transferred into 48-well plates at confluent cell density (25,000 cells/cm^2^). On the second day after plating, the culture medium was replaced with osteogenic differentiation medium (StemPro Osteogenesis Differentiation kit, Gibco). 10 nM PTH or vehicle (PBS) was added into the cultures. PTH treated cells received intermittent PTH stimulation by adding PTH (10 nM) to the culture medium for 1 h every day, then the medium was replaced with fresh osteogenic differentiation medium to wash out the PTH. Cells were cultured in this way for 14 d and then osteogenic differentiation was analyzed by staining with Alizarin red S (Sigma). To quantitate Alizarin red staining, the stained cells were rinsed with PBS, and extracted with 10% (wt/vol) cetylpyridinium chloride in 10 mM sodium phosphate (pH 7.0) for 10 min at room temperature. Alizarin red in the extract was quantitated by optical density measurement at 562 nm.

### Western Blots.

Primary bone marrow stromal cells were transferred into 48-well plates at confluent cell density (25,000 cells/cm^2^). On the second day after plating, the culture medium was replaced with osteogenic differentiation medium (StemPro Osteogenesis Differentiation kit, Gibco). Next, 10 nM PTH, 10 ng/mL SOSTi, 10 µM Wnt agonist (Santa Cruz), or vehicle were then added. To intermittently stimulate with PTH, PTH was added to the cultures (10 nM) for 1 h every day, then the medium was replaced with fresh osteogenic differentiation medium to wash out the PTH. Prior to extracting proteins, cells were washed with PBS and then lysis buffer was added (50 mM Tris⋅HCl, 150 mM NaCl, 1% Nonidet P-40, 0.5% sodium deoxycholate, 0.1% SDS, 1 mM sodium vanadate, 0.5 mM sodium fluoride, and cOmplete Mini EDTA-free Protease Inhibitor Mixture [Sigma]). The cells were scraped off the plate in the lysis buffer, transferred to an Eppendorf tube on ice, incubated for 20 min with occasional vortexing, then centrifuged at 17,000 × *g* for 10 min at 4 °C to clear cellular debris. The cell lysates were Western blotted with the indicated antibodies and immunoreactive bands were detected using ECL reagent (Pierce). Antibodies included anti–β-catenin (D10A8), anti–β-actin (D6A8), anti-–SK-3β (27C10), antiphospho-GSK-3α/β (Ser21/9, 9331S), anti-Smad1 (D5907), antiphospho-Smad1 (Ser206; D40B7), anti-P38 MAPK (D13E1), antiphospho-p38 MAPK (Thr180/Tyr182; D3F9), and HRP-linked anti-rabbit IgG secondary antibody, all from Cell Signaling. We also used anti-mouse osteolectin antibody (AF3729) and HRP-linked anti-goat IgG secondary antibody from R&D Systems. Band intensities on Western blots were quantitated using ImageJ.

### Reverse-Transcription Quantitative PCR.

Bone marrow stromal cells were washed with PBS and lysed using TriReagent (Sigma). RNA was extracted and reverse transcribed into cDNA using iScript RT (oligo dT and random priming; Bio-Rad). Quantitative PCR was performed using the CFX384 Real-Time System (Bio-Rad). The primers used for qPCR analysis of mouse RNA included: *Osteolectin*: 5′-AGG TCC TGG GAG GGA GTG-3′ and 5′-GGG CCT CCT GGA GAT TCT T-3′; *Actb*: 5′-GCT CTT TTC CAG CCT TCC TT-3′ and 5′-CTT CTG CAT CCT GTC AGC AA-3′; *Lef1*: 5′-TGT TTA TCC CAT CAC GGG TGG-3′ and 5′-CAT GGA AGT GTC GCC TGA CAG-3′; *Runx2*: 5′-TTA CCT ACA CCC CGC CAG TC-3′ and 5′-TGC TGG TCT GGA AGG GTC C-3′; *Alpl*: 5′-CCA ACT CTT TTG TGC CAG AGA-3′ and 5′-GGC TAC ATT GGT GTT GAG CTT TT-3′.

### Cell Imaging.

To quantify live or dead cells in cultures of bone marrow stromal cells, cells were washed with 1× HBSS and stained with the LIVE/DEAD Cell Imaging Kit (488/570) (Invitrogen, R37601). Cells were then imaged using a Zeiss LSM880 confocal microscope and analyzed using Zeiss Zen-2.

### Statistical Methods.

In each type of experiment, multiple mice were tested in multiple independent experiments performed on different days. Mice were allocated to experiments randomly and samples processed in an arbitrary order, but formal randomization techniques were not used. Prior to analyzing the statistical significance of differences among treatments, we tested whether the data were normally distributed and whether variance was similar among treatments. To test for normal distribution, we performed the Shapiro–Wilk test when 3 ≤ *n* < 20 or the D’Agostino Omnibus test when *n* ≥ 20. To test if variability significantly differed among treatments, we performed Levene’s median tests. When the data significantly deviated from normality or variability significantly differed among treatments, we log_2_-transformed the data and tested again for normality and variability. If the transformed data no longer significantly deviated from normality and equal variability, we performed parametric tests on the transformed data. If log_2_-transformation was not possible or the transformed data still significantly deviated from normality or equal variability, we performed nonparametric tests on nontransformed data.

We used two-sided statistical tests where applicable (see figure legends for details). All statistical analyses were performed with GraphPad Prism 9.0.0. All data represent mean ± SD. Samples sizes were not predetermined based on statistical power calculations but were based on our experience with these assays.

## Supplementary Material

Supplementary File

## Data Availability

All study data are included in the article and *SI Appendix*.
