# Supplementary material for: The effect of parathyroid hormone on osteogenesis is mediated partly by osteolectin
Source: Proc Natl Acad Sci U S A. 2021 Jun 21;118(25):e2026176118. doi: 10.1073/pnas.2026176118 (PMC8237660; doi:10.1073/pnas.2026176118)
Supplement: Supplementary File [file pnas.2026176118.sapp.pdf]

PNAS
www.pnas.org

Supplementary Information for

The effect of Parathyroid hormone on osteogenesis is mediated partly by Osteolectin

Jingzhu Zhang, Adi Cohen, Bo Shen, Liming Du, Alpaslan Tasdogan, Zhiyu Zhao, Elizabeth J. Shane, and Sean J. Morrison.

Sean J. Morrison
Email: Sean.Morrison@UTSouthwestern.edu

This PDF file includes:

Figures S1 to S3

Supplemental Figure 1

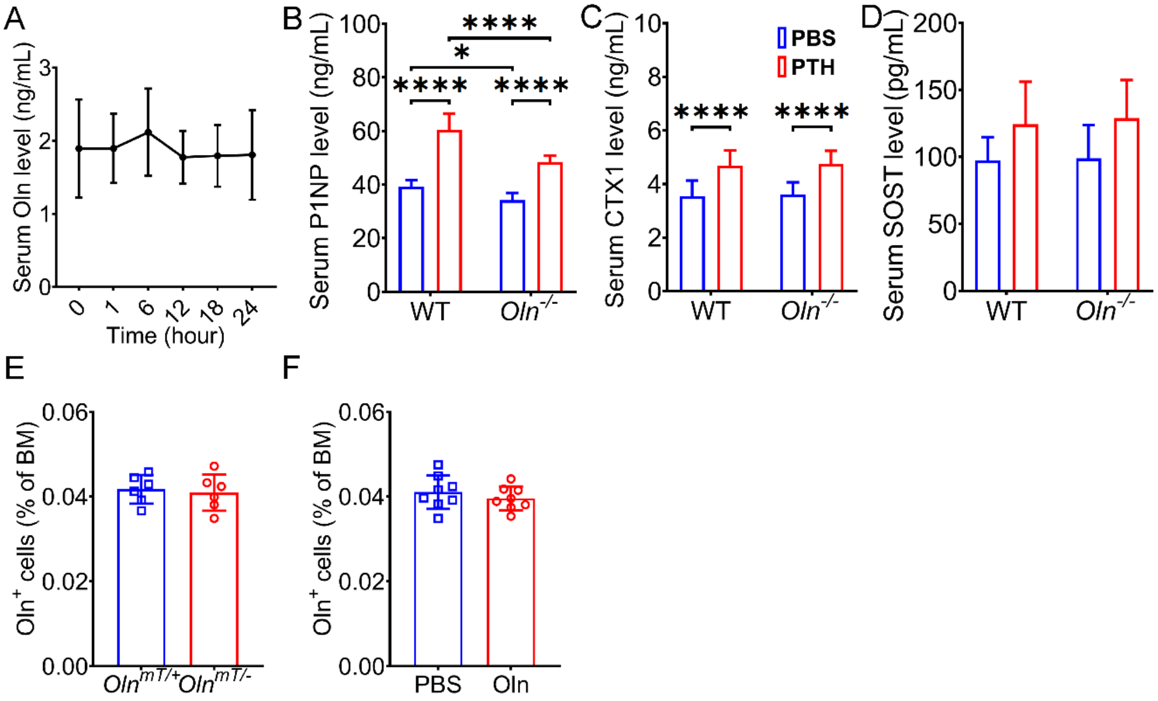

Fig. S1. Osteolectin contributes to the effect of PTH on bone formation but not bone resorption. (A) Serum Osteolectin levels were measured in female wildtype mice 0.5, 1, 2, 4, 8, and 24 hours after subcutaneous injection with PTH (n=5 mice per time point). (B-D) Two-month old Osteolectin deficient (Oln-/-) and littermate control (WT) mice were treated with PTH daily for four weeks, then serum P1NP levels (B), serum CTX1 levels (C) and serum SOST levels (D) were measured (n = 5 males and 5 females in each group). (E) We observed no effect of Osteolectin deficiency on the frequency of Osteolectin+ stromal cells (rapidly dividing osteogenic progenitors, (5)) in the bone marrow of OsteolectinmTomato/+ (OlnmT/+) and OsteolectinmTomato/- (OlnmT/- ; Osteolectin deficient) mice (n = 3 male and 3 female mice per group). (F) We observed no effect of treatment with recombinant Osteolectin for four weeks on the frequency of Osteolectin+ stromal cells in the bone marrow of OlnmT/+ mice (n = 4 male and 4 female mice per group). All data represent mean ± SD. Statistical significance was assessed using one-way ANOVAs followed by Dunnett's multiple comparisons tests (A), or two-way ANOVAs followed by Sidak's multiple comparisons tests (B-D), or unpaired t tests (E and F). * P < 0.05; **** P < 0.0001.

Supplemental Figure 2

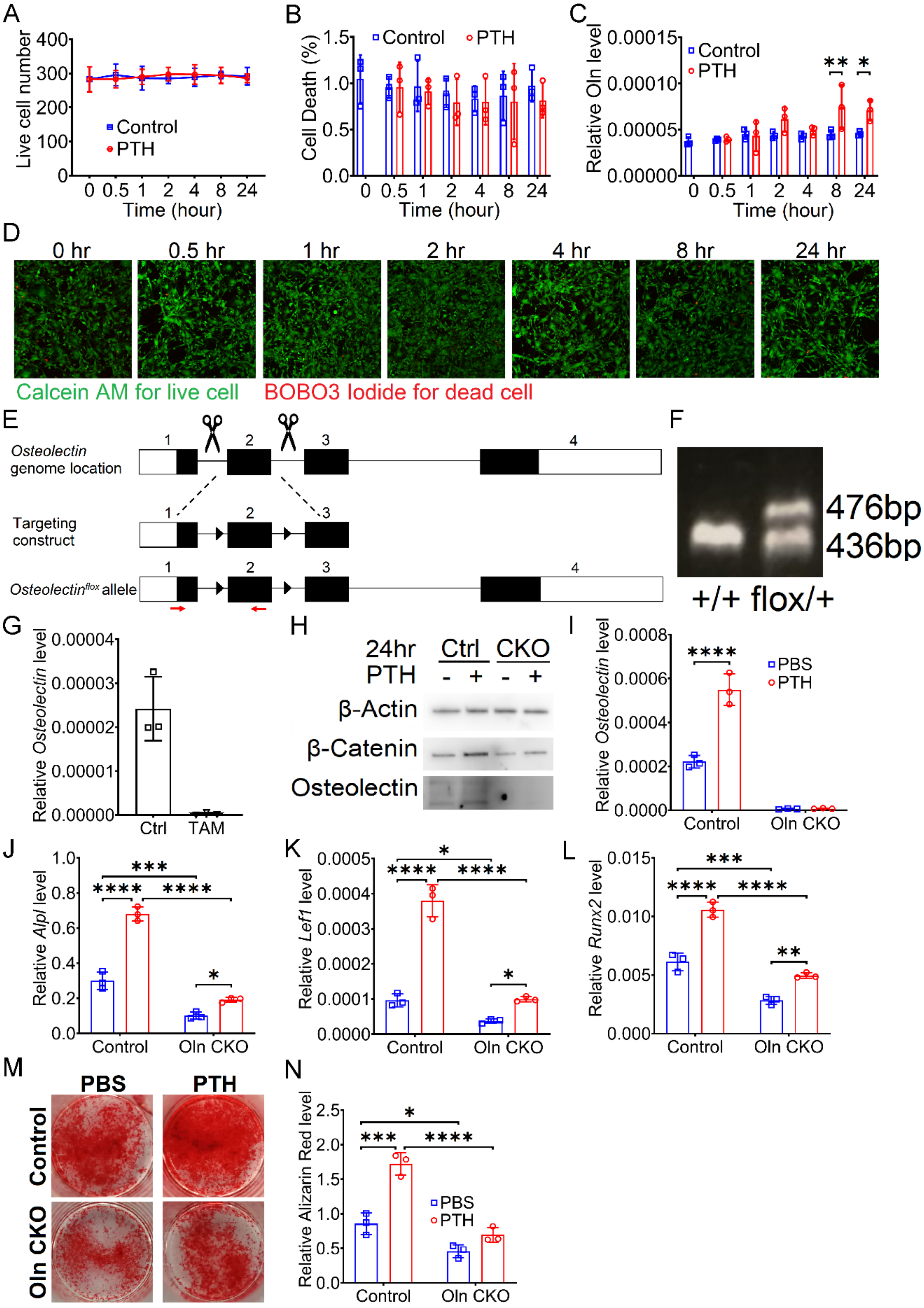

Fig. S2. Osteolectin contributes to the effect of PTH on Wnt pathway activation. (A-D) Bone marrow stromal cells were cultured in osteogenic differentiation medium with intermittent PTH treatment for 0.5, 1, 2, 4, 8, or 24 hours. At each time point, the numbers of live and dead cells were counted using Calcein AM (green) and BOBO3 Iodide (red), respectively. (C) Osteolectin transcript levels were analyzed by RT-qPCR (n = 3 independent experiments). (E) We generated an Osteolectinflox allele by inserting loxP elements (black arrows) on either side of exon2 of Osteolectin. Deletion of exon2 leads to a frame shift that would be expected to give a complete loss of Osteolectin function (13). The loxP insertion sites were chosen to avoid disrupting sequences conserved among species. Scissors indicate the positions of sequences targeted by sgRNAs. Red arrows indicate the locations of genotyping primers. The gene targeting was performed in C57BL/Ka zygotes and then mice were backcrossed at least three times onto a C57BL/Ka background before analysis. (F) PCR genotyping demonstrated germline transmission of the Osteolectinflox allele. (G) RT-qPCR analysis of Osteolectin transcript levels in whole bone marrow cells from UBCCreER/+;Osteolectinflox/flox mice treated for three days with 2mg/mouse/day of tamoxifen (TAM) or vehicle control (n = 3 mice per group). (H-N) Primary bone marrow stromal cells from UBCCreER/+;Osteolectinflox/flox mice were cultured adherently and treated with 200nM 4-hydroxytamoxifen for two days. (H) These cells were then transferred into osteogenic differentiation medium, treated with PBS or intermittent PTH (10nM), and then lysed 24 hours later and immunoblotted for β-actin, β-catenin, and Osteolectin (n = 3 independent experiments). Other cultures were lysed three days later and analyzed by RT-qPCR to assess Osteolectin transcript levels (I) and Wnt target gene transcript levels including Alpl (J), Lef1 (K) and Runx2 (L) (n = 3 independent experiments). (M, N) Bone marrow stromal cells cultured in osteogenic differentiation medium were treated with intermittent PTH or PBS for 14 days and stained with Alizarin red S (n = 3 independent experiments). All data represent mean ± SD. Statistical significance was assessed using paired samples two-way ANOVAs followed by Dunnett's (treatment comparisons of A-C, I-L and N) or Holm-Sidak's (genotype comparisons of J-L and N) multiple comparisons tests. * $P < 0.05$; *** $P < 0.001$; **** $P < 0.0001$.

Supplemental Figure 3

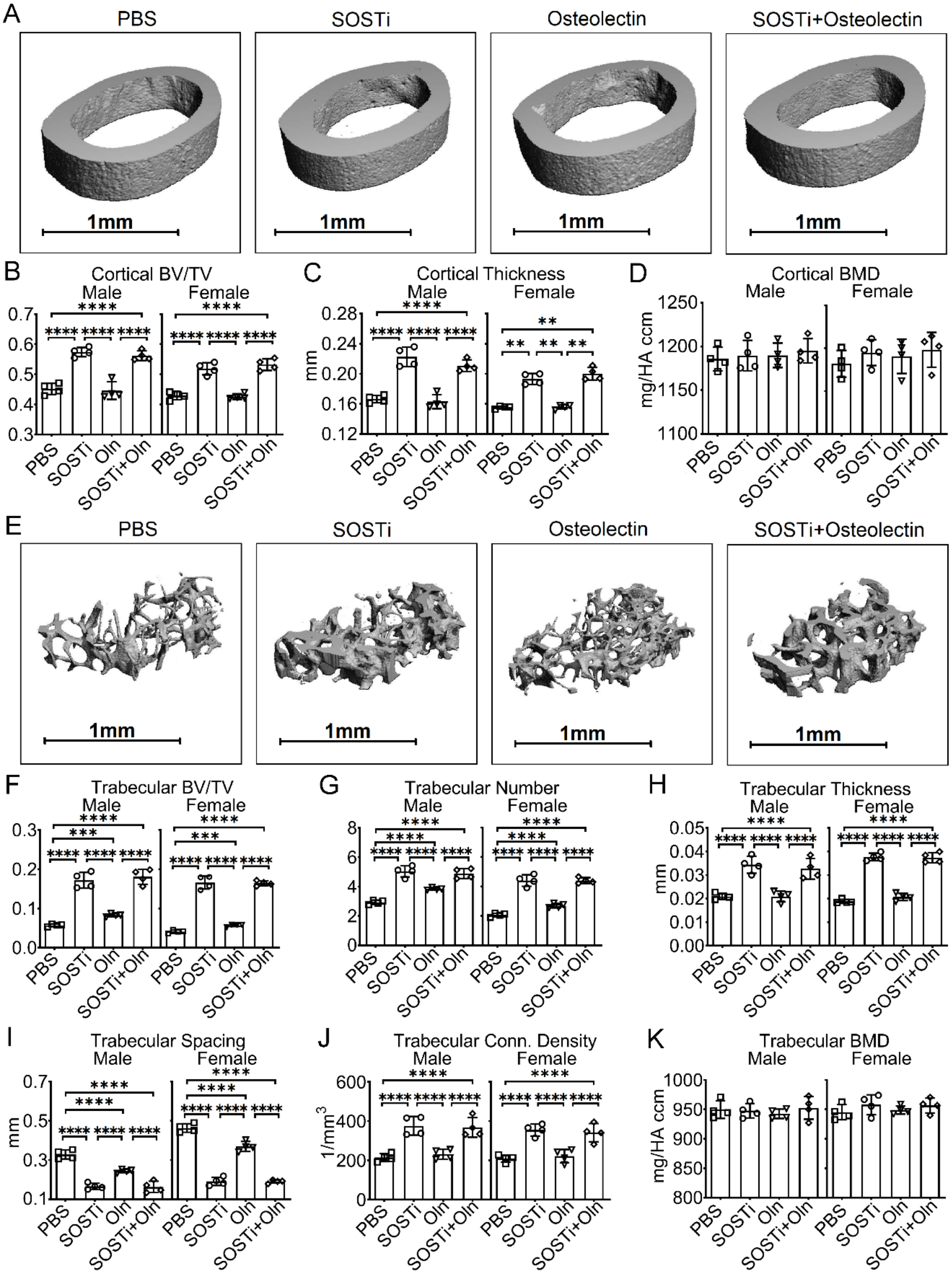

Fig. S3. Combinatorial treatment with SOSTi plus Osteolectin did not have additive effects. (A) Representative microCT images of cortical bone in the mid-femur diaphysis of male wildtype mice treated with PBS, SOSTi, Osteolectin, or the combination of Osteolectin plus SOSTi for one

month. (B-D) MicroCT analysis of the cortical bone volume/total volume (B), cortical thickness (C), and cortical bone mineral density (D) in the mid-femur diaphysis. (E) Representative microCT images of trabecular bone in the distal femur metaphysis of male mice. (F-K) MicroCT analysis of trabecular bone volume/total volume (F), trabecular number (G), trabecular thickness (H), trabecular spacing (I), trabecular connectivity density (J), and trabecular bone mineral density (K) in the distal femur metaphysis. For all experiments, n = 4 mice per treatment group. All data represent mean ± SD. Statistical significance was assessed using one-way ANOVAs (C and F) or two-way ANOVAs (B, D, and G-K) followed by Tukey's multiple comparisons tests (** $P < 0.01$; *** $P < 0.001$; **** $P < 0.0001$).
